# The Risk of Some Veterinary Antimicrobial Agents on Public Health Associated with Antimicrobial Resistance and their Molecular Basis

**DOI:** 10.3389/fmicb.2016.01626

**Published:** 2016-10-18

**Authors:** Haihong Hao, Pascal Sander, Zahid Iqbal, Yulian Wang, Guyue Cheng, Zonghui Yuan

**Affiliations:** ^1^China MOA Laboratory for Risk Assessment of Quality and Safety of Livestock and Poultry Products, Huazhong Agricultural UniversityWuhan, China; ^2^National Reference Laboratory of Veterinary Drug Residues (HZAU) and MOA Key Laboratory for the Detection of Veterinary Drug Residues in Foods, Huazhong Agricultural UniversityWuhan, China; ^3^Laboratory of Fougères, French Agency for Food, Environmental and Occupational SafetyFougères Cedex, France

**Keywords:** antimicrobial agents, food-producing animal, antimicrobial resistance, public health, molecular basis

## Abstract

The risk of antimicrobial agents used in food-producing animals on public health associated with antimicrobial resistance continues to be a current topic of discussion as related to animal and human public health. In the present review, resistance monitoring data, and risk assessment results of some important antimicrobial agents were cited to elucidate the possible association of antimicrobial use in food animals and antimicrobial resistance in humans. From the selected examples, it was apparent from reviewing the published scientific literature that the ban on use of some antimicrobial agents (e.g., avoparcin, fluoroquinolone, tetracyclines) did not change drug resistance patterns and did not mitigate the intended goal of minimizing antimicrobial resistance. The use of some antimicrobial agents (e.g., virginiamycin, macrolides, and cephalosporins) in food animals may have an impact on the antimicrobial resistance in humans, but it was largely depended on the pattern of drug usage in different geographical regions. The epidemiological characteristics of resistant bacteria were closely related to molecular mechanisms involved in the development, fitness, and transmission of antimicrobial resistance.

## Introduction

Antimicrobial agents have been used in food animal production since the 1950s. Antimicrobial agents have contributed significantly to the prevention and treatment of infectious diseases in food animals and some of them have played a very important role in the promotion of animal growth and feed efficiency (Dibner and Richards, 2005; Niewold, 2007). Since many classes of antimicrobial agents used in food animals are also used in human medicine, there is the potential for selection, and spread of antimicrobial resistant bacteria in animals to humans through the food supply. Human health consequences have been raised concerning whether the use of antimicrobial agents in food animals may minimize the effectiveness of the same classes of medically important antimicrobial agents to treat antimicrobial resistant infectious diseases in humans (Salisbury et al., 2002). In this respect, the administration of low doses (5–40 mg/kg.feed) of antimicrobial growth-promoters in animal feed were banned by Europe (EU) in 2006 to protect public health, and this ban drew a great attention of other countries and international organizations (Marshall and Levy, 2011) since this low dose drug exposure over a long period of time could elicit selective pressure leading to the emergence of resistant bacteria.

For comprehensive surveillance monitoring of antimicrobial resistance in food-borne pathogens, many countries have established antimicrobial resistance monitoring systems, such as the National Antimicrobial Resistance Monitoring System (NARMS) in United States of America (USA) and Danish Integrated Antimicrobial Resistance Monitoring and Research Program (DANMAP). The Food and Agriculture Organization (FAO), World Health Organization (WHO), and World Organization for Animal Health (OIE) have organized expert workshops on the risk assessment and management of non-human antimicrobial usage and their resistance(FAO/OIE/WHO, 2003, 2004, 2006). WHO/FAO/OIE have jointly carried out systematic evaluation of veterinary antimicrobial resistance for the impact on public health (FAO/OIE/WHO, 2006). The European Medicines Agency (EMA), European Food Safety Authority (EFSA) and European Center for Disease Prevention and Control (ECDC) have also work together for the Joint Interagency Antimicrobial Consumption and Resistance Analysis (JIACRA) and have also published reports on antimicrobial use in food animals and antimicrobial resistance recently (ECDC/EFSA/EMA, 2015).

In the present review, results of risk assessments based on data from different antimicrobial resistance monitoring systems are reported to evaluate the stewardship programs of antimicrobial use in food animals. Resistance to some representative antimicrobial agents (e.g., cephalosporins, tetracyclines, fluoroquinolones, macrolides, glycopeptides, and streptogramins) in some selected pathogens (e.g., *Enterococci spp, Campylobacter spp, Salmonella spp*, and *Escherichia coli*) were taken as examples to describe the relationship between antimicrobial resistance and drug usage in food animals. The molecular mechanism involved in the development, fitness, and transmission of antimicrobial resistance was also integrated into the review to provide a comprehensive understanding of the antimicrobial resistance and to identify the need for risk management of antimicrobial drugs.

## Avoparcin and glycopeptide-resistant enterococci

Avoparcin, a vancomycin analog, was effective against gram-positive bacteria by disturbing their cell wall synthesis. Avoparcin had been widely used as a feed additive in food animals during 1940s–1990s. As a member of glycopeptides, there was concern that the misuse of avoparcin may confer cross-resistance to glycopeptides and in particular vancomycin which is known as one of the important last-line antimicrobials in human medicine. In 1993, isolation and frequency of vancomycin-resistant *Enterococci* (VRE) from food-producing animals in Great British drew public health concern about the consequences of wide use of avoparcin as a growth promoter in animals. From 1995 to 2000, Denmark, United Kingdom, EU members, Japan and China gradually banned the use of avoparcin in food-producing animals.

It is important to keep in mind that the term of VRE includes several combinations of bacterial species (e.g., *Enterococcus faecium and Enterococcus faecalis*) and resistance genes (*vanA, B, C, D, E, G, L, M*, and *N*). The characteristics of *van A-N* genes are summarized in Table 1. The *vanA, B, D, E, G, L, M*, and *N* are acquired genetic determinants in *E. faecium* and/or *E. faecalis*, while *vanC* is intrinsic gene present in *E. gallinarum* and *E. casseliflavus*/*E. flavescens* (Cetinkaya et al., 2000; Boyd et al., 2008). Among those, *E. faecium* with *vanA* type vancomycin resistance is clinically most important because *vanA* is an acquired and transferable gene which is resistant to both vancomycin and teicoplanin (Nilsson, 2012). The *vanB* and *vanN* are transferable but is susceptible to teicoplanin (Nomura et al., 2012). The *vanM* is newly found in China and confirmed to have transferability and resistance to both vancomycin and teicoplanin (Xu et al., 2010; Chen et al., 2015).

**Table 1:** **Characteristics of glycopetide-resistant genes in Enterococci**.

**Characteristics**	***vanA***	***vanB***	***vanC***	***vanD***	***vanE***	***vanG***	***vanL***	***vanM***	***vanN***
**Enterococcus species**	***E. faecium; E. faecalis***	***E. faecium; E. faecalis***	***E. gallinarum, E. casseliflavus, E. flavescens***	***E. faecium***	***E. faecalis***	***E. faecalis***	***E. faecalis***	***E. faecium***	***E. faecium***
Vancomycin MIC (*μ*g/mL)	64–1024	4–1024	2–32	16–64	16–32	16–32	8	>256	16
Teicoplanin MIC (*μ*g/mL)	16–512	< 1	< 1	2–16	< 0.5	< 0.5	< 0.5	96	0.5
Genetic determinant	acquired	acquired	intrinsic	acquired	acquired	acquired	acquired	acquired	acquired
transferable	Yes	Yes	No	No	No	No	No	Yes	Yes
New precursor of ligase	D-ala-D-lac	D-ala-D-lac	D-ala-D-ser	D-ala-D-lac	D-ala-D-ser	D-ala-D-ser	D-ala-D-ser	D-ala-D-lac	D-ala-D-ser
Expression	Inducible	Inducible	constitutive	constitutive	Constitutive or inducible	Constitutive or inducible	Constitutive or inducible	Inducible	constitutive

Extensive use of avoparcin for animal growth promotion in most parts of Europe may be the reason for high prevalence of VRE in the intestinal microbiota of farm animals in Europe during the 1990s (Aarestrup, 1995; Klare et al., 1995). Once the use of avoparcin was prohibited, prevalence of VRE among farm animals decreased in some EU countries. According to DANMAP report, substantial reductions (from 80 to 0%) in the prevalence of VRE were observed between 1995 and 2013, after the ban on avoparcin as growth promoter in Denmark (DANMAP, 2013). Very few vancomycin resistant enterococci have been isolated from Danish livestock and produced meat during 2003–2013 (DANMAP, 2013).

However, it was noteworthy that ban on avoparcin did not effectively reduce incidence of vancomycin-resistant *E. faecium* in avian feces. A paper published in 2008 showed that vancomycin-resistant *E. faecium* was still highly prevalent in poultry in Europe (Werner et al., 2008). Even after 15 years of the EU ban on avoparcin, vancomycin-resistant *E. faecium* was still present in the food chain and could be detected in 47% of the broiler feces (Garcia-Migura et al., 2007; DANMAP, 2010). The proportion of broilers colonized with vancomycin-resistant *E. faecium* increased from less than 1% in 2000 to over 40% in 2005, even though Sweden had forbidden the use of avoparcin as growth promoter since 1986 (Nilsson et al., 2009). The high prevalence of vancomycin-resistant *E. faecium* in EU countries may be due to the transferability of *E. faecium* with the *vanA* gene (Nilsson et al., 2009). The *vanN* gene with transferability was also found in vancomycin-resistant *E. faecium* isolated from chicken meat (Nomura et al., 2012).

The most important concern is not only that VRE are present among farm animals but also their potential to transfer resistance genes to vancomycin susceptible enterococci and other Gram—positive bacteria that may be transmitted via food products to humans. Some earlier reports showed that hospital isolates of *E. faecium* generally clustered in subgroups which were different from those found in animals (Top et al., 2004; Willems et al., 2005). In contrast, some in *vivo* transfer studies indicated that *vanA* gene was located in transposon Tn1546 and may be transferred between animal and human adapted enterococci (Jensen, 1998; Lester et al., 2006; Lester and Hammerum, 2010). The *vanM* gene was also located in transferable element and could transfer by conjugation (Xu et al., 2010). Similar strains of VRE have been isolated from both farm animal and human, indicating that some of those strains may adapt to farm animals and cause infectious diseases in humans (Freitas et al., 2011). However, the origin of Tn1546 element encoding *vanA* resistance in US hospitals was still unknown (Jensen, 1998; Lester et al., 2006; Lester and Hammerum, 2010). It was difficult to determine to what extent did the presence of VRE among farm animals actually affect public health (Nilsson, 2012).

Notably, a controversy was perceived in the geographical distribution of VRE among humans. In the United States, about 20,000 (or 40%) of *Enterococcus* healthcare-associated infections in 2013 were vancomycin resistant, including 77% of vancomycin resistant *E. faecium* (CDC, 2013). In Denmark, vancomycin resistance was detected in only 3.4% of *E.faecium* isolates from bloodstream infections in 2013 (DANMAP, 2013). In other Nordic countries, the level of vancomycin resistant *Enterococcus* was even lower than in Denmark in recent years (EARS-Net, 2012). Therefore, VRE was more common in US hospitals than that in European hospitals, although in the USA, avoparcin had never been approved for use in food animals (Gambarotto et al., 2000; Bonten et al., 2001). It was likely that the serious problem of VRE in US hospitals was not related to the use of avoparcin in food animals but to that of vancomycin use in human medicine, because therapeutic vancomycin treatment was much higher in the USA when compared with that in Europe (Acar et al., 2000).

## Virginiamycin and streptogramin-resistant *Enterococci*

Virginiamycin is a streptogramin antimicrobial which could block the transpeptidation or translocation of protein synthesis in bacteria. It has been used for the prevention of *Clostridial enteritis* and enhancement of growth and feed efficiency in poultry, swine and cattle for more than 30 years in Japan, Canada, U.S.A, and other countries. However, EU prohibited its use in food-producing animals in 1999 because it was assumed to select for streptogramin-resistant *Enterococci* (SRE) and lead to treatment failure of patients in hospitals with pristinamycin and Synercid (Quinupristin-Dalfopristin) resistant *Enterococcus faecium* infections. Mechanisms conferring resistance to streptogramin in *E. faecium* was mediated via related acetyltransferases (VatD and VatE), erythromycin ribosomal methylase B (ErmB) and staphylococcal-type lactonase (VgbA) (Werner et al., 2002).

Some epidemiological investigations showed that streptogramin-resistant genes (*vat*) were detected in 25% of virginiamycin-resistant *E. faecium* isolated from pigs and chickens, and in 29% of isolates from farm workers in Denmark (Hammerum et al., 1998; Haroche et al., 2000). The NARMS report revealed that long-term use of virginiamycin for growth promotion was likely to result in the emergence of streptogramin- resistant *Enterococcus* which was present in 30–70% of poultry products purchased from supermarkets (NARMS, 2012).

Transferability of these *vat* genes was observed among *E. faecium* isolates from food-animals (Sørensen et al., 2001). The resistant strains may also spread indirectly to human beings from farms through the environment including raw manure or surface/ground water (Smith et al., 2003). Sørensen et al. (2001) found that streptogramin- resistant *E. faecium* from food animals was able to establish transient populations in the gut after experimental ingestion. Additionally, similar genetic patterns of *vanA* containing *Enterococci* isolates, from poultry and human, were reported in Spain (Robredo et al., 2000).

However, streptogramin-resistant *E. faecium* from poultry may be well-adapted to cloaca, but difficult to survive against the gastric barrier and colonization resistance in the gut (Smith et al., 2003). Despite the high rate of exposure in contaminated meat, prevalence of streptogramin resistant *E. faecium* remained low and therefore, bacteria with high resistance could rarely establish in human beings (McDonald et al., 2001). By using pulsed-field gel electrophoresis (PFGE) technique, Hershberger et al. (2005) found distinct gene cluster of streptogramin-resistant *E. faecium*, isolated from poultry and human beings. Additionally, numerous studies found the different genetic profiles of streptogramin- resistant *E. faecium*, isolated from food-animals (poultry and pork) and human beings, indicating that the bacterial strains might be highly host specific (Smith et al., 2003; Hershberger et al., 2005; Hammerum et al., 2010).

The US Food and Drug Administration and Center for Veterinary Medicine (FDA-CVM) reported that there was not enough data to show the transmission of SRE between animals and humans (FDV-CVM, 2004). A quantitative risk assessment performed by US FDA-CVM concluded that risk of SRE, caused by virginiamycin use in food animals, was about 1–14 heads per billion due to the treatment failure of VRE infections (FDV-CVM, 2004). A quantitative human health risk and benefits assessment for virginiamycin showed that human health benefits of virginiamycin withdrawal ranged from zero to less than one statistical life and withdrawal of the drug may cause more human illnesses than it would prevent (Cox and Popken, 2004; Cox, 2005). Phillips (2007) showed that human health risk from resistance among enterococci selected by virginiamycin was small (Phillips, 2007). Some scientists have disagreed with the conclusion that the ban of virginiamycin in EU could lead to an increased prevalence of streptogramin resistant enterococci (Hammerum et al., 2007). Although an investigation showed that the continued use of virginiamycin as growth promoter in poultry may increase the potential for streptogramin- resistant *E. faecium* infection in humans (Kieke et al., 2006).

## Fluoroquinolone and resistance in *Campylobacter*

Fluoroquinolones, a family of synthetic broad-spectrum antimicrobial agents, played an important role in the treatment of bacterial infection in both veterinary medicine and in human medicine. Fluoroquinolones can inhibit the DNA synthesis of bacteria by selectively inhibiting their DNA gyrase and/or topoisomerase (Suto et al., 1992). The fluoroquinolone resistance in Campylobacter was mediated by mutations in the drug target enzyme (e.g., Thr-86-Ile mutation in GyrA) and/or by overexpression of efflux pumps (e.g., CmeABC) (Griggs et al., 2005).

Both enrofloxacin and ciprofloxacin were the second generation of fluroquinolones. They had similar structural and antimicrobial activity. The enrofloxacin was approved to treat bacterial infections in poultry in the USA before 2005, while ciprofloxacin was used in human medicine to treat foodborne infections such as *Campylobacter, Salmonella, E.coli*, and *Shigella*. When enrofloxacin was administrated into some food producing animal, it could be metabolized to ciprofloxacin (Gratacós-Cubars´i et al., 2007). The close relationship between fluoroquinolone drug in veterinary medicine and it use in human medicine may raise the risk of fluoroquinolone resistance from animal to human.

There was evidence that the use of enrofloxacin in poultry production would induce fluoroquinolone resistance in *Campylobacter jejuni* and these fluoroquinolone resistant bacteria transferred to humans and contributed to the treatment failure of campylobacterosis in humans via poultry exposure (FDA, 2002; Nelson et al., 2007). Some in *vitro* and in *vivo* studies have demonstrated that FQ-resistant strains would rapidly emerge when *Campylobacter* was exposed to FQs (e.g., enrofloxacin). The frequencies of emergence may range between approximately 10^−6^–10^−8^/cell/generation in culture media, indicating that resistant bacteria would inevitably emerge when cell population was sufficiently larger than 10^6^ (Yan et al., 2006; Han et al., 2008). FQ-susceptible *C. jejuni* in chicken could rapidly attain FQ-resistance within 24 h after the initiation of treatment with enrofloxacin (McDermott et al., 2002; Luo et al., 2003; van Boven et al., 2003; Farnell et al., 2005; Griggs et al., 2005). The FQ-resistant *Campylobacter* population could eventually colonize into intestinal tract of birds and may be transmitted to human via the contaminated poultry meat (Luangtongkum et al., 2009).

Due to above reasons, US FDA withdrew the use of enrofloxacin in poultry in 2005 (USFDA, 2005). After withdrawal of enrofloxacin from poultry, the rate of FQ-resistance in *C. jejuni* decreased in chicken during 2005–2007 (NARMS, 2010). Human clinicians also observed a reduction in domestically acquired *Campylobacter* infections with decreased susceptibility to fluoroquinolones, and it was thought to be a great achievement regarding public health (Nelson et al., 2007). However, during 2008–2011, the positive rate of ciprofloxacin-resistant *C. jejuni* from retail chicken was again increased (14.6–22.7%) in the USA (NARMS, 2012). These studies suggest that the policy on the ban of fluoroquinolone use in poultry did not reduce or eliminate reservoirs of FQ -resistant *C. jejuni* with subsequent reemergence and persist in poultry products in the USA.

The spread of FQ-resistant *C. jejuni* in USA might result from high mutation rate and enhanced fitness of the bacteria in chicken reservoirs (Luangtongkum et al., 2009). Previous studies have demonstrated that resistant *C. jejuni*, carrying Thr-86-Ile mutation in GyrA, could colonize chicken caecum in the absence of antimicrobial selection pressure (Luo et al., 2005; Nelson et al., 2007; Han et al., 2012; Zeitouni et al., 2012). The Thr-86-Ile mutation in GyrA can modulate DNA supercoiling homeostasis and result in better survivability of FQ-resistant *C. jejuni* in chicken host (Han et al., 2012). Due to the enhanced fitness, it will be difficult to reduce the prevalence of FQ-resistance in *C. jejuni*, even though farmers have not used these antimicrobials in poultry. Additionally, the NARMS human data showed that ciprofloxacin resistant *C. jejuni* in USA kept increasing from 16.7 in 1997 to 25.3% in 2012 (NARMS, 2012).

Contrary to the situation in USA, the ECDC/EFSA/EMA JIACARA reported data showed no associations between the consumption of fluoroquinolones in food-producing animals and the occurrence of resistance in *Campylobacter spp* from cases of human infection (ECDC/EFSA/EMA, 2015). Although the growth promoting agents of fluoroquinolone were withdrawn in European countries earlier in the Twenty first century, the incidence of FQ -resistant *C. jejuni* in broilers raised from 5.3 in 2001 to 26% in 2013 (DANMAP, 2013). The relationship between fluorquinolone use in food animals and antimicrobial resistance in humans may be different based on the geographic region, poultry production environment and fluoquinolone surveillance monitoring protocols.

## Macrolides and resistance in *Campylobacter*

Macrolides inhibit bacteria protein biosynthesis by preventing peptidyltransferase location and/or inhibiting ribosomal translation (Tenson et al., 2003; Liang and Han, 2013). Macrolides were the first choice for treatment infections caused by Gram-positive bacteria in animal and campylobacterosis in human. Macrolide resistance develops in *Campylobacter* by point mutation in target genes of 23S rRNA, ribosomal protein and by overexpression of efflux pumps (Hao et al., 2013).

In food-producing animals, macrolide drugs, such as tylosin and tilmicosin, have been used as growth promoters for decades in USA and Canada. However, two macrolide members, tylosin, and spiramycin were banned for their use as animal growth promoters in Finland and EU since 1995 because exposure of animals to these drugs was implicated as a possible cause of treatment failure in *Campylobacter* infections in humans.

From the ECDC/EFSA/EMA JIACARA report, positive associations were noted for total consumption of macrolides in food-producing animals in 2011 and 2012 and the occurrence of resistance in *C. jejuni* from cases of human infection (ECDC/EFSA/EMA, 2015). However, the data obtained from European antimicrobial resistance monitoring systems, like DANMAP, revealed that macrolide resistance in *C. jejuni* isolates from Danish broilers kept at a relatively low level (lower than 1%) and there was no significant temporal variation during 2000–2012 (DANMAP, 2013).

Contrary to the ECDC/EFSA/EMA JIACARA report, the USA risk assessment data showed that tylosin and tilmicosin in food animals did not result in a risk to public health in relationship to the development and dissemination of macrolide resistant *Campylobacter* (Hurd et al., 2004). The probability of treatment failure of drug resistant *Campylobacter* infections was only one person in 2.36, 14, and 53 billion per year due to consumption of beef, poultry, and pork, respectively (Hurd et al., 2004). In other words, the probability of therapy failure of human campylobacteriosis due to resistant bacteria in food animals exposed to tylosin or tilmicosin, was much less than the mortality due to automobile accidents (1/7,000), shootings (1/10,000), motorcycle and car accidents (1/500,000), aircraft accidents (1/1,000,000), lightning strikes (1/3,000,000) and shark attacks (1/100,000,000) in USA (Hurd et al., 2004). Therefore, the two veterinary macrolide drugs (tylosin and tilmicosin) may have negligible risk for human health (Casewell et al., 2003; Phillips et al., 2004a,b; Turnidge, 2004). Data obtained from NARMS also revealed that macrolide resistance in *C. jejuni* isolates from American chicken breast kept at a relatively low level (lower than 1%) and there was no significant temporal variation during 2000–2012 (NARMS, 2012; DANMAP, 2013).

The mutation frequency for macrolide resistance in *Campylobacter* was reported to be about 10^−10^/cell/generation which is approximately 10,000-fold lower than that of FQ resistance (Yan et al., 2006; Lin et al., 2007). The mutants obtained by single-step selection tend to have low-to-intermediate levels of macrolide resistance (Erythromycin MIC = 8–64 μg/ml) (Kim et al., 2006; Lin et al., 2007; Caldwell et al., 2008). These mutants generally had fitness cost in the absence of macrolide drugs (Kim et al., 2006; Caldwell et al., 2008). Acquisition of mutations in 23S rRNA, which conferred a high level of resistance to erythromycin (MIC ≥ 512 μg/ml), appeared to require stepwise selection and/or prolonged exposure to macrolide drugs (Lin et al., 2007; Caldwell et al., 2008). In the absence of macrolide selection pressure, most of the 23S rRNA mutations could be stably maintained without competition (Gibreel et al., 2005; Caldwell et al., 2008), but due to their fitness burden, they could be rapidly outcompeted by the erythromycin susceptive *C. jejuni* in both *vitro* or *vivo* environment (Hao et al., 2009; Almofti et al., 2011a,b; Luangtongkum et al., 2011; Zeitouni et al., 2012).

Since *Campylobacter coli* is the most common *Campylobacter* species in pigs, we also discuss the topic of macrolide resistant *C. coli* in this review. The ban on tylosin as growth promoter had a remarkable effect on the level of erythromycin resistance in *C. coli* from pigs, as it decreased from 66 to 20% in Denmark between 1998 and 2005 (Hammerum et al., 2007). However, DANMAP data showed that during 2006–2010, the macrolide resistance in *C. coli* varied within the range of 10–20% without significant reduction (DANMAP, 2010). The macrolide resistance in *C. coli* isolates, although more prevalent than that in *C. jejuni* isolates, also did not increase over the past 10 years since the beginning of the monitoring in the U.S.A (NARMS, 2010). A previous study demonstrated that *C. coli* were not intrinsically more mutable than *C. jejuni*, because no elevated mutation frequency for erythromycin was observed in *C. coli* (Lin et al., 2007).

## Tetracyclines and resistance in *Salmonella*

Tetracyclines are protein synthesis inhibitors. They bind to the 30S ribosomal subunit and prevent the binding of aminoacyl-tRNA to mRNA-ribosome complex (Bassetti et al., 2013). Bacteria could develop resistance to tetracycline by efflux pumps (TetA, B, C, D, E, F, G, H, I, J, K, L, P(A), P(B),V, Y, Z, 30, OtrB, TcrC), ribosomal protection (TetM, O, Q, S, T, U, W, OtrA,), and enzymatic inactivation of drugs (TetX) (Linkevicius et al., 2015). Moreover, the tetracycline resistance genes are often located in some transferable elements including plasmids and integrons (Szmolka et al., 2015).

Tetracyclines have widely been used in human and veterinary medicine since the first discovery of tetracycline in 1948. In food animal production, tetracyclines, like oxytetracycline, and chlorotetracycline, were broadly used for growth promotion and prophylaxis (Chopra and Roberts, 2001). The data from antimicrobial resistance monitoring programs showed that tetracycline resistance was commonly detected in foodborne pathogens (DANMAP, 2014; EFSA, 2015). EU countries have banned the use of tetracyclines for growth promotion since 2006.

However, withdrawal of tetracycline growth promoters did not alter the epidemiology of tetracycline resistance in EU counties. DANMAP reported that tetracycline resistant *Salmonella* Typhimurium isolated from pigs had increased from less than 30% in 2001 to 47% in 2013 (DANMAP, 2013). It was also stated that 29,797 kg (active compound) of tetracyclines were sold to the pig industry in Denmark during 2013 and total consumption of the drugs in pig industry was increased by 2-fold in 2013 as compared to that in 2001 (DANMAP, 2013). The increased therapeutic use of tetracyclines might be a primary reflection of the increased occurrence of drug resistance in *S*. Typhimurium and *E.coli* isolates from pigs (DANMAP, 2010). Using logistic regression analysis, the results from a previous study also showed that both the *S*.Typhimurium phage type (*p* < 0.0001) and the increase in tetracycline consumption (*p* = 0.0007) were significantly associated with the antimicrobial resistance (Emborg et al., 2007). The ECDC/EFSA/EMA JIACARA reported positive associations for total consumption of tetracyclines and the occurrence of resistance in *Salmonella spp* from cases of human infection in 2011 and 2012 (ECDC/EFSA/EMA, 2015). Additionally, tetracycline-resistant *S*. Typhimurium also became more prevalent in human cases and reported as domestically acquired sporadic (36%) and outbreak related (21%) (Emborg et al., 2007).

The high prevalence of tetracycline resistance in zoonotic pathogens could be explained by the transfer of already established or new resistant clones rather than the conversion of well-established susceptible clones into resistant ones by uptake of resistance genes (Szmolka et al., 2015). The serotype of *Salmonella* may also decide the prevalence of resistance to tetracylines. It is known that *Salmonella* Enteritidis and *S*. Typhimurium are two general serotypes of *Salmonella* associated with public health. However, the resistance to tetracycline in *S*. Enteritidis (5%) from human was much lower than that in *S*. Typhimurium (DANMAP, 2010). The drug resistance among *S*. Typhimurium isolates might be due to the transferrable ability of tetracycline resistance determinants (e.g., class 1 integron and *Salmonella* genomic island 1) and the possible fitness of these determinants in pig host (Anjum et al., 2011).

## Cephalosporins and resistance in *Salmonella* and *E. coli*

The cephalosporins are a class of β-lactams which can disrupt the synthesis of the peptidoglycan layer forming the bacterial cell wall. Cephalosporins have great significance in the treatment of bacterial infection in human medicine. The third and fourth generation cephalosporins are the most common antimicrobial drugs used as human medicine worldwide. However, the third generation cephalosporins, such as ceftiofur, has also been extensively used in many different food animals and the fourth-generation cephalosporin, like cefquinome, was approved by the US Food, and Drug Administration (US FDA) in 2007 soon after its approval by the European Union.

Gram-negative bacteria may develop resistance to β-lactams by producing β-lactamase to inactivate the drugs. The major public health concern is that use of third and fourth generation of cephalosporins in food animals might result in resistance development in foodborne pathogens (e.g., *Salmonella* and *E. coli*). Some evidence, provided by Keep Antibiotic Working (KAW), Union of Concerned Scientists (UCS), the American Medical Association (AMA) and the Infectious Diseases Society of America (IDSA), showed that approval of cefquinome might induce the development of resistance in foodborne pathogens and enhance the transfer risk of resistance to human which may compromise public health. Recently the FDA ordered to prohibit extra label use of cephalosporin drugs in food-producing animals (FDA, 2015).

Interestingly, NARMS data showed that there was no considerably change of cephalosporins resistance in *E. coli* and *Salmonella* isolates from ground turkey, ground beef and pork chop during 2002–2012 (NARMS, 2012). Although there was a transient increase in cephalosporin resistance of *Salmonella* from ground turkey during years of 2007–2009, yet it decreased again in the year 2012. During 2002–2012, antimicrobial resistance was increased (10–35%) in *Salmonella* isolates from chicken breast and the resistance rate was considerably lower in *E. coli* isolates than that in *Salmonella* isolates. In the case of *E. coli* from chicken breast, ground turkey and pork chop, resistance was lower than 10% (except for 11.7% in chicken breast in 2009) and even less than 1% for the ground beef bacteria from 2002 to 2010 (NARMS, 2010).

Additionally, the NARMS human data showed that cephalosporins resistance in *Salmonella* and *E.coli*O157 kept at a very low level (less than 1%) during 2000–2012 (NARMS, 2012). From the ECDC/EFSA/EMA JIACARA report, no associations were observed between the consumption of 3rd—and 4th—generation cephalosporins in food-producing animals and the occurrence of resistance to this sub-class in selected bacteria from human (ECDC/EFSA/EMA, 2015).

Concern of resistant bacteria carrying extended-spectrum β-lactamases (ESBLs) has been raised after the use of third and fourth generation of cephalosporins. The ESBLs encoding genes (e.g., *bla*OXA, *bla*PSE, *bla*SHV, *bla*TEM, *bla*CTX-M), as well as the plamid-mediated AmpC β-lactamases (PMAβ, such as *bla*CMY and *bla*FOX), and carbepenemases (e.g., *bla*IMP, *bla*KPC, *bla*VIM) could be involved in the resistance to extended-spectrum β–lactams (Liebana et al., 2013; Rubin and Pitout, 2014; Bae et al., 2015). There were many reports about the transmission of ESBL carrying bacteria (Hasman et al., 2005; Collignon and Aarestrup, 2007), but the possible zoonotic spread of ESBL is still controversial. Some investigators found that there was similarity between ESBL genes and bacterial properties in isolates from human, livestock and companion animal populations, indicating that exchange of ESBL genes and ESBL bacterial between these reservoirs (Valentin et al., 2014; Dahms et al., 2015). A review paper by Ewers et al. (2012) showed that the European, American and Asian countries (e.g., Japan) shared a similar population of ESBL subtypes, but ESBL subtype (*bla*_*CTX*−*M*_) from human was similar to that from pets but significantly different with that from food-producing animals (Ewers et al., 2012). Largely unknown environmental factors might impact the spread of resistant pathogens and increase the complexity of development and transfer of resistance enzymes. For example, wild animals like waterfowl, prey and rodents carry similar subtype of ESBL *E. coli* to humans, indicating that wildlife could be an environmental reservoir and melting pot for enzymatic resistance. The bacteria might re-infect humans through the omnipresent bird feces. Similar to humans traveling, the birds migration might also contribute to the worldwide spread of the resistant organism (Guenther et al., 2009, 2010, 2011; Dolejska et al., 2011).

## Conclusions

The relationship between use of antimicrobial agents in food animals and antimicrobial resistance associated with human public health is a complex and controversial subject (Table 2). The risk of the use of some antimicrobial agents in food-producing animals with consequences on human public health is still problematic because there are so many factors to consider from an antimicrobial resistance perspective (Table 2). There is not enough of compelling evidence to assert that the prevalence of some resistant bacteria in humans was due to antimicrobial agents used in food animals (NAMI, 2010; Horigan et al., 2016). The zoonotic spread of antimicrobial resistant bacteria or resistant genes is also questionable because resistant pathogens could be found in soil, water and environment and long-term occurrence of antimicrobial resistant genes in nature was even known before the antimicrobial era (Casewell et al., 2003; Aminov and Mackie, 2007; Kobayashi et al., 2007; Aminov, 2010). Therefore, it is not wise to oversimplify the opinion that the resistant bacteria from food producing animal is a major origin of human infection and neglect the highly complex environment scenario.

**Table 2:** **Summary of risk assessment of some veterinary antimicrobial drugs on human public health associated with antimicrobial resistance and their molecular basis**.

**Veterinary drug Use and ban**	**Associated Public health**	**Resistance monitoring data in animasl**	**Resistance monitoring data in humans**	**Risk assessment; Risk association**	**Molecular basis**
Avoparcin, Used 1940s–1990s; Banned 1995–2000; Not approved in USA for use in animal.	Vancomycin-resistant *Enterococci* (VRE), and *E. faecium* (VREF)	In EU, high prevalence of VRE in 1990s and in poultry after 2000; In Denmark, VRE reduced from 1995 to 2013; few VRE in livestock during 2003–2013.	In U. S. A, 40% VRE infections in 2013; In EU, < 5% VRE in 2013.	Positive risk; Still some controversy questions	vanA gene located in transferable transposon Tn1546
Virginiamycin, Used as GP for 30 years; Banned in 1999 in EU	*streptogramin-resistant Enterococci (SRE)*, and *E.faecium* (SREF)	In Denmark, 25% SREF from pigs and chickens; In USA, 30–70% SREF from poultry products in 2012.	Very rare in human hospital.	FDA-CVM: risk is little weight	VatD, VatE; ErmB; VgbA; Hard resistance development
Veterinary fluoroquinolones, e.g., enrofloxacin Banned use on poultry in 2005 in USA	fluoroquinolones resistant *Campylobacter jejuni*	In USA, high prevalence of FQ-resistant *C. jejuni* in poultry before 2005, resistance reduced during 2005–2007, resistance increased during 2008–2011.	In USA, ciprofloxacin resistant *C. jejuni* kept increasing from 16.7% in 1997 to 25.3% in 2012.	FDA-CVM: Positive risk ECDC/EFSA/EMA JIACARA: no risk associations	Thr-86-Ile mutation in GyrA; high mutation rate and enhanced fitness in chicken
Veterinary Macrolides e.g., tylosin, tilmicosin. EU banned tylosin and spiramycin as GP since 1995	Macrolide resistant *Campylobacter spp*	Resistant *C.jejuni* kept low level (< 1%) in USA and Denmark; Resistant *C. coli* in pig reduced during 1998 –2005 and kept at about 10% during 2006–2010 in Denmark; kept at a stable level in USA in the past decade.	Erythromycin resistant *C. jejuni* is rare in human	FDA-CVM: negative risk ECDC/EFSA/EMA JIACARA: positive risk associations	point mutation in target genes of 23S rRNA; low mutation frequency and fitness cost of resistance
Veterinary tetracyclines EU banned tetracyclines as growth promotor since 2006	Tetracycline resistance in *Salmonella* Typhimurium	In Denmark, resistant *S*.Typhimurium from pigs had increased from less than 30% in 2001 to 47% in 2013.	High prevalent of tetracycline-resistant *S*. Typhimurium in human	ECDC/EFSA/EMA JIACARA report: positive associations	*Tet* genes were normally located in some transferable elements
Veterinary Cephalosporins	Cephalosporins resistance in *E. coli* and *Salmonella*	In USA, no significant change of resistance from animal product during 2002–2012;	Resistance kept at a very low level (< 1%) during 2000–2012 in USA.	ECDC/EFSA/EMA JIACARA report: no risk associations	Complex distribution of ESBLs in animal, human and environment.

*GP was Growth promotor; ESBL was extended-spectrum β-lactamases; ECDC/EFSA/EMA JIACARA was European center for disease prevention and control/European food safety authority/European Medicines Agency. Joint Interagency Antimicrobial Consumption and Resistance Analysis report. Risk association means the association between consumption of veterinary antimicrobial drugs in food-producing animal and the occurrence of resistance bacterial from human infection. The FDA-CVM risk means the relationship between the use of antimicrobial agents in food-producing animal and human public health associated with antimicrobial resistance in special foodborne pathogens*.

Furthermore, the ban on some antimicrobial usage has not altered or decreased the incidence of resistance in foodborne pathogens. This may be due to the enhanced fitness or high transferability of some resistant determinants. To control the increased animal disease, therapeutic levels of some antimicrobial drugs (e.g., fluroquinolones and tetracyclines) has been increased. This may also be a reason for the increased detection of resistance to some therapeutic drugs in food borne pathogens isolated from food animals after the ban of growth promoters (Koluman and Dikici, 2013).

On the concept of “one health one world,” international governments need to cooperate to establish an international antimicrobial resistance surveillance monitoring program and monitor the antimicrobial resistance trends in human and animals for a long time. Both the benefit and risk outcomes should be considered into the risk assessment and management. To find wise strategy to control antimicrobial resistance, it is necessary to considerate the chemotherapeutic medicine, microbiology and agricultural environment and fully understand molecular basis involved in the emergence of antimicrobial resistance.

## Author contributions

The Idea and designation: HH, and ZY; Data collection and analysis: HH, PS, and YW; The writing and modification of this manuscript: HH, ZI, PS, and GC.

## Funding

This article is financially supported by grants from National Basic Research Program of China (2013CB127206), National Key Research and Development Program (2016YFD0501302), Fundamental Research Funds for the Central Universities (2662015PY035), Morning Program of Wuhan in China (2015070404010191), National Program for Risk Assessment of Quality and Safety of Livestock and Poultry Products (GJFP2016008), National Natural Science Foundation of China (31101856/31302143/31272614), National Key Technology R&D Program (2012BAK01B00).

### Conflict of interest statement

The authors declare that the research was conducted in the absence of any commercial or financial relationships that could be construed as a potential conflict of interest.
